# Supplementary material for: Polymorphisms in Pfkelch13 domains before and after the introduction of artemisinin-based combination therapy in Southwest Nigeria
Source: PLoS One. 2025 Mar 31;20(3):e0316479. doi: 10.1371/journal.pone.0316479 (PMC11957316; doi:10.1371/journal.pone.0316479)

Samples: 13716
Bases: 1155
Average spacing: 12

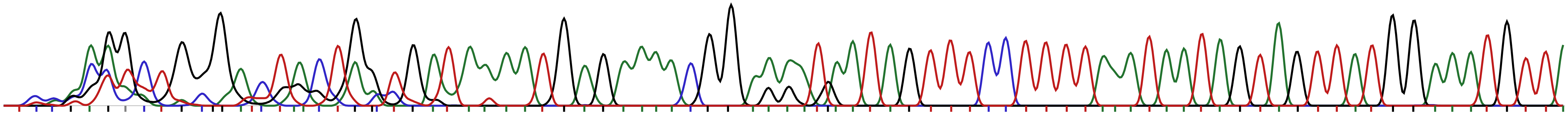

10 20 30 40 50 60 70 80

T C C G A G - C T G CGG ATC TAA C T G G C T G A T A A A A T G A G A A A A C G G A A A A T G A A T A G T T T C C T T T T A A A T A A T A G T A G T T A T G G A A A T G T T A

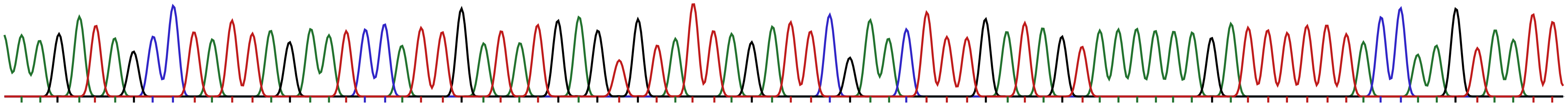

90 100 110 120 130 140 150 160 170

A A G A T A G C C T A T T A G A A T C C A T T G A T A T G A G T G T A T T A G A T T C G A A C T T T G A T A G T A A A A A A G A T T T T T T A C C A A G T A A T T

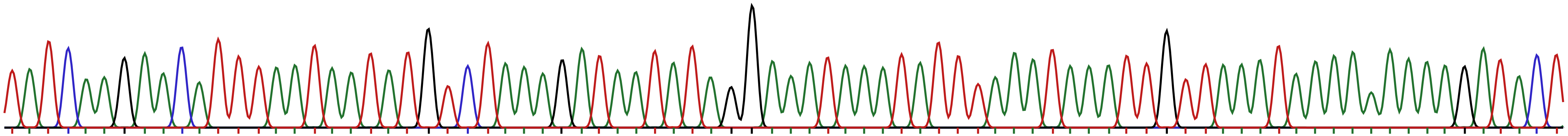

180 190 200 210 220 230 240 250

T A T C A A G A A C A T T T A A T A A T A T G T C T A A A G A T A A T A T A G G A A A T A A A T A T T T A A A T A A A T T G T T A A A T A A A A A A A A G A T A C T

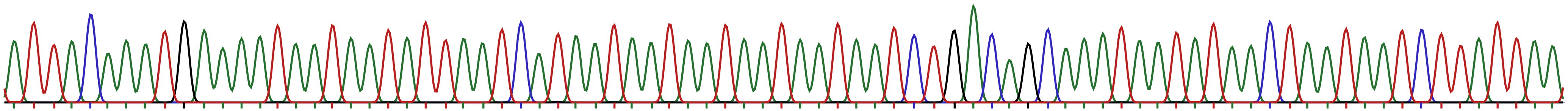

260 270 280 290 300 310 320 330

A T T A C A A A T G A A A A T A A T A A T A T T A A T C A T A A T A A T A A T A A T A A T A A T C T G A C A G C A A A T A A T A T A A C T A A T A A T C T T A T T A A

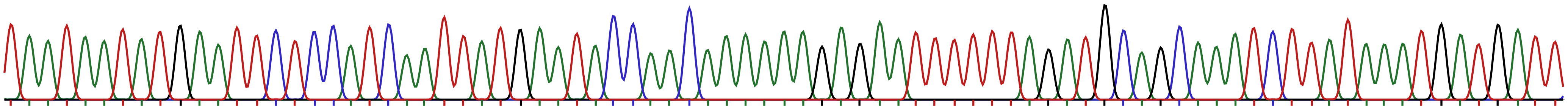

340 350 360 370 380 390 400 410

T A A T A A T A T G A A T T C T C C A T C A A T T A T G A A T A C C A A C A A A A A A G A G A A T T T T T T A G A T G C A G C A A A T C T T A T A A A T G A T G A T T

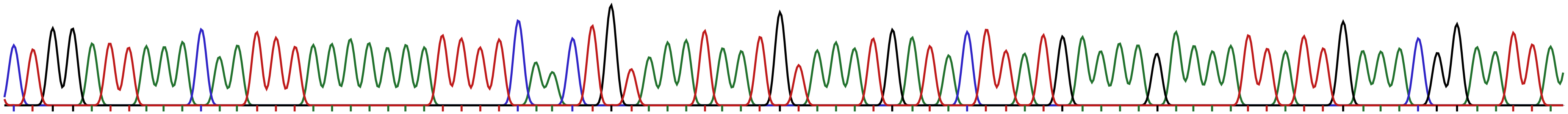

420 430 440 450 460 470 480 490 500

C T G G A T T A A A C A A T T T A A A A A A A A T T T T C A A C T G T A A A T A A T G T A A A T G A T A C T T A T G A A A A G A A A A T T A T T G A A A C G G A A T T A

Samples: 13716
Bases: 1155
Average spacing: 12

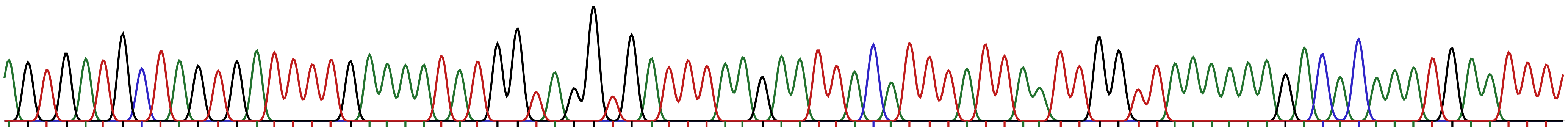

510 520 530 540 550 560 570 580
A G T G A T G C T A G T G A T T T T G A A A A T A T G G T A G G T G A T T T A A G A A T T A C A T T T A T T A A T T G G T T A A A A A A G A C A C A A A T G A A T T T

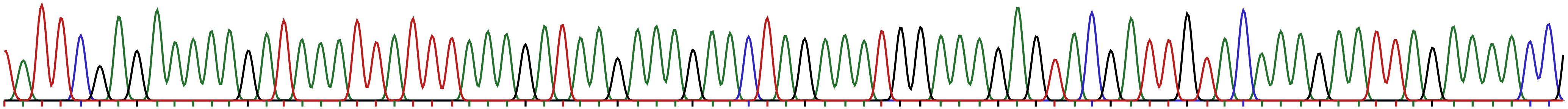

590 600 610 620 630 640 650 660
T A T T C G A G A A A A A G A T A A A T T A T T T A A A G A T A A G A A A G A A C T A G A A A T G G A A A G A G T A C G A T T G T A C A A A G A A T T A G A A A A C C

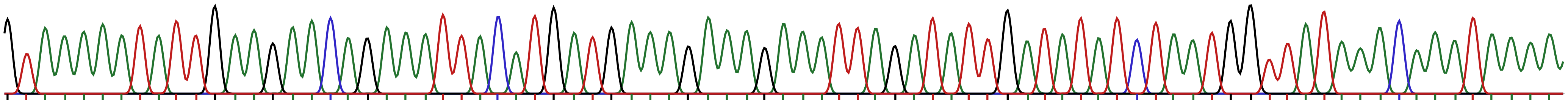

670 680 690 700 710 720 730 740 750
G T A A A A A T A T T G A A G A A C A G A A A T T A C A T G A T G A A A G A A A G A A A T T A G A T A T T G A T A T A T C T A A T G G T T A T A A A C A A A T A A A A

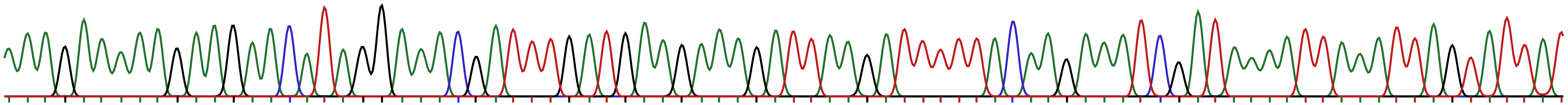

760 770 780 790 800 810 820 830
A A A G A A A A A G A A G A A C A T A G G A A A C G A T T T G A T G A A G A A A G A T T A A G A T T T T T A C A A G A A A T C G A T A A A A T T A A A T T A G T A T T A T

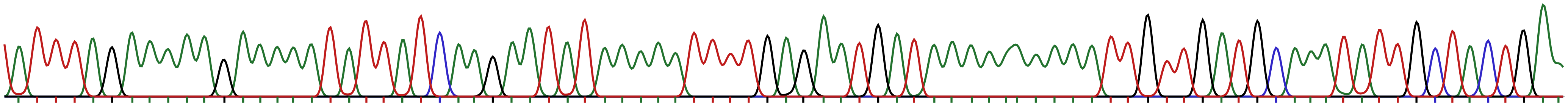

840 850 860 870 880 890 900 910 920
A T T T A G A A A A A G A A A A A T A T T A T C A A G A A T A T A A A A A T T T T G A G A A T G A T A A A A AA A A A A T T G T T G A T G C A A A T A T T G C T A C T G A A

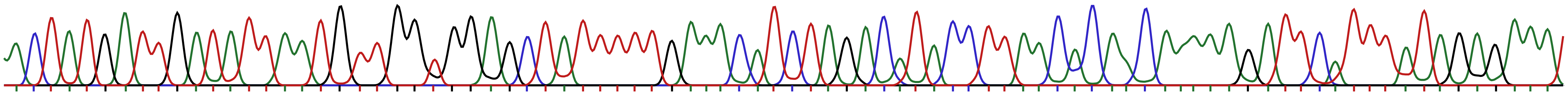

930 940 950 960 970 980 990 1000
A C T A T G A T T G A T A T T A A T G T T G G T G G A G C T A T T T T T G A A A C A T C T A G A C A T A C C T T A A C A C A A C A A A A A G A T T C A T T T A T A G A G A A A

Samples: 13716
Bases: 1155
Average spacing: 12

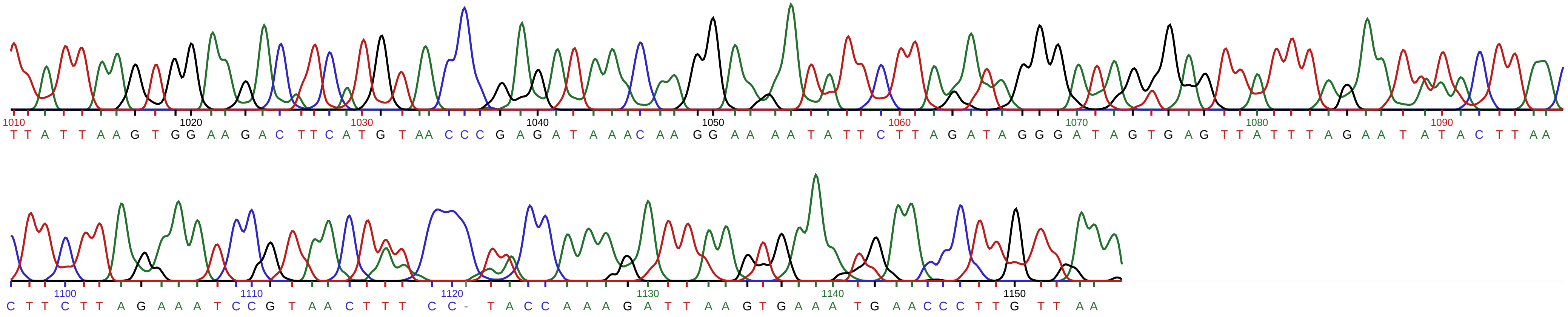

Supplement: Supporting information 2 — (ZIP) [file pone.0316479.s002.zip › 042KN2F_PREMIX_Plate_KELCH1_H09.pdf]
